# Detecting Abnormality of Battery Lifetime from First‐Cycle Data Using Few‐Shot Learning

**DOI:** 10.1002/advs.202305315

**Published:** 2023-12-11

**Authors:** Xiaopeng Tang, Xin Lai, Changfu Zou, Yuanqiang Zhou, Jiajun Zhu, Yuejiu Zheng, Furong Gao

**Affiliations:** ^1^ Dept. Chemical and Biological Engineering Hong Kong University of Science and Technology Clear Water Bay Kowloon Hong Kong SAR 999077 China; ^2^ Science Unit Lingnan University Tuen Mun Hong Kong SAR 999077 China; ^3^ School of Mechanical Engineering University of Shanghai for Science and Technology Shanghai 200093 China; ^4^ Department of Electrical Engineering Chalmers University of Technology Gothenburg 41296 Sweden; ^5^ Guangzhou HKUST Fok Ying Tung Research Institute Guangzhou Guangdong 511458 China

**Keywords:** big data, early‐stage detection, few‐shot learning, lifetime abnormality, lithium‐ion battery

## Abstract

The service life of large battery packs can be significantly influenced by only one or two abnormal cells with faster aging rates. However, the early‐stage identification of lifetime abnormality is challenging due to the low abnormal rate and imperceptible initial performance deviations. This work proposes a lifetime abnormality detection method for batteries based on few‐shot learning and using only the first‐cycle aging data. Verified with the largest known dataset with 215 commercial lithium‐ion batteries, the method can identify all abnormal batteries, with a false alarm rate of only 3.8%. It is also found that any capacity and resistance‐based approach can easily fail to screen out a large proportion of the abnormal batteries, which should be given enough attention. This work highlights the opportunities to diagnose lifetime abnormalities via “big data” analysis, without requiring additional experimental effort or battery sensors, thereby leading to extended battery life, increased cost‐benefit, and improved environmental friendliness.

## Graphical abstract

The lifetime of large battery packs can be influenced by only one or two abnormal cells with faster aging rates in it. This work proposes a method to predict battery lifetime abnormality using only the first‐cycle battery aging data and achieves a typical accuracy >90%. It can be used to screen out abnormal batteries before grouping.

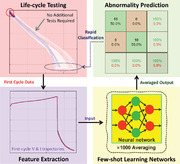

## 1 Introduction

The lithium‐ion battery is widely regarded as a promising device for achieving a sustainable society.^[^ ^1^, ^2^ ^]^ Nevertheless, its manufacturing process is always accompanied by high consumption of energy and raw materials.^[^ ^3^, ^4^ ^]^ Therefore, a long enough service life is critical to achieve net‐zero carbon emissions and make positive environmental impacts,^[^ ^5^, ^6^ ^]^ especially noting that the electricity for battery manufacturing and charging is mostly (70−−80% for the globe) generated from fossil fuels today.^[^ ^7^, ^8^ ^]^

The long service life of individual batteries may not necessarily guarantee the satisfactory life‐cycle performance of a battery pack, in which hundreds of battery cells are connected in series and parallel to meet the power and energy requirements of applications such as electric vehicles and renewable energy storage.^[^ ^9^ ^]^ Without proper maintenance, the service life of a large battery pack can be significantly reduced by only one or two abnormal cells with faster aging rates,^[^ ^10^, ^11^, ^12^ ^]^ even if the majority of the candidates in the pack have normal aging behaviors. Screening out these few batteries with abnormal lifetime performances prior to battery grouping and pack assembly can improve the capacity, lifetime, and cost‐benefit of a battery pack with immediate effect.^[^ ^13^ ^]^

Many previous studies have emphasized battery screening and their core idea is to group batteries with similar key parameters into a pack. To date, the most widely used screening method in the industry is the capacity‐resistance (CR) method,^[^ ^14^ ^]^ in which batteries with similar capacity and resistance values are assumed to have similar performance. In addition to these two indexes, incremental capacity peaks,^[^ ^15^ ^]^ pulse charging responses,^[^ ^16^ ^]^ voltage trajectories,^[^ ^17^ ^]^ and electrochemical impedance spectroscopy^[^ ^18^ ^]^ have also been applied for battery screening. These tests are fast, requiring no more than 12 h in general. In addition, they can effectively screen out the abnormalities that are immediately observable, e.g., high resistance. However, the lifetime abnormality, involving the long‐term decay of the battery's future capacity, is not considered.

Identifying the batteries' lifetime abnormality is challenging, especially at the beginning of their service life.^[^ ^19^ ^]^ First, abnormal aging behaviors are more likely to be perceived in the latter part of the battery life, compared to much less information to be possibly extracted in the first few cycles.^[^ ^20^ ^]^ As a result, even the best existing algorithms still need to use the data collected from the first 3–5 cycles of the aging process for abnormality detection.^[^ ^21^, ^22^ ^]^ In addition to the increased testing time, their prediction errors are also as large as 10 to 15%. Second, the low abnormal rate itself poses challenges to dataset establishment.^[^ ^23^, ^24^ ^]^ To collect sufficient abnormal samples, we have to carry out long‐term aging tests on a large number of batteries, making the experiments costly and time‐consuming. Finally, the correctness of a classification (made at the beginning stage of the battery life) can only be experimentally verified after long‐term battery usage. The delayed feedback hinders the algorithm's development. With these issues in mind, the early‐stage identification of the battery lifetime abnormality remains an unsolved problem in the field of battery manufacturing and management.

In this work, we make the first attempt to identify the lifetime abnormality of lithium‐ion batteries using only the first‐cycle aging data. A few‐shot learning network is developed to detect the lifetime abnormality, without requiring prior knowledge of degradation mechanisms. We generate the largest known dataset for lifetime‐abnormality detection, which contains 215 commercial lithium‐ion batteries with an abnormal rate of 3.25%. Our method can accurately identify all abnormal batteries in the dataset, with a false alarm rate of only 3.8%. The overall accuracy achieves 96.4%. In addition, we find that the widely used capacity‐resistance‐based methods are not suitable for identifying lifetime abnormality, which must draw enough attention from the battery community. Our proposed identification algorithm offers a reliable and cost‐effective way to immediately improve the lifetime of multi‐cell battery packs, without requiring additional experimental effort, battery sensors, or knowledge of aging mechanisms, thereby leading to extended battery life, increased cost‐benefit, and improved environmental friendliness.

## 2 Results and Discussion

### 2.1 Data Generation

A group of 215 commercial batteries have been tested in this work (type: 18650, chemistry: LiNi_0.8_Co_0.1_Mn_0.1_O_2_/graphite). These batteries underwent sequentially an initial resistance test, an initial capacity test, and an accelerated aging test with the current rate increased to 3C. During accelerated aging, seven out of 215 batteries exhibited abnormal aging behaviors. The generated dataset is shared publicly for further battery research and development, as described in Data Availability Statement Section. The full experimental details are provided in Supporting Information.

### 2.2 Limitations of the Capacity‐Resistance Method

**Figure 1:**
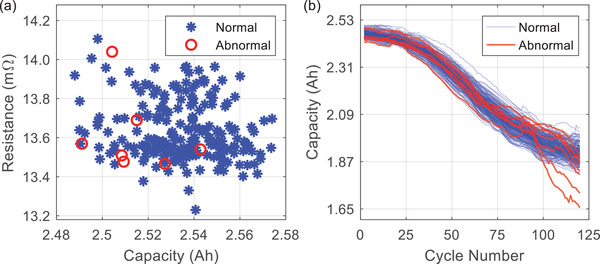
Illustration of our battery aging data. a) Initial resistance versus capacity of 215 batteries. b) Capacity degradation trajectories of 215 batteries. Here, the batteries aging at an average rate of lower than 10 mAh/cycle after the 90^th^ cycle are labeled with “normal”, while the others are labeled with “abnormal”.

We first check the initial capacity and resistance of all the batteries, with the results shown in **Figure** **1**. All the 215 batteries share a similar initial capacity (2.53 ± 0.05 Ah) and resistance (13.7±0.5 mΩ). However, their aging trajectories, as shown in Figure 1b, can be highly different even if these batteries underwent the same aging test. To be specific, in the last 25% of the aging test (90^th^ to 120^th^ cycle), seven out of the 215 batteries exhibit a significantly faster local aging rate (>10 mAh/cycle on average) than the others (<5 mAh/cycle). These seven batteries are, therefore, defined as “abnormal”. From the data monitoring point of view, these abnormal samples are also defined as “positive samples”, while the normal batteries are termed as “negative samples” in the following discussions.

**Figure 2:**
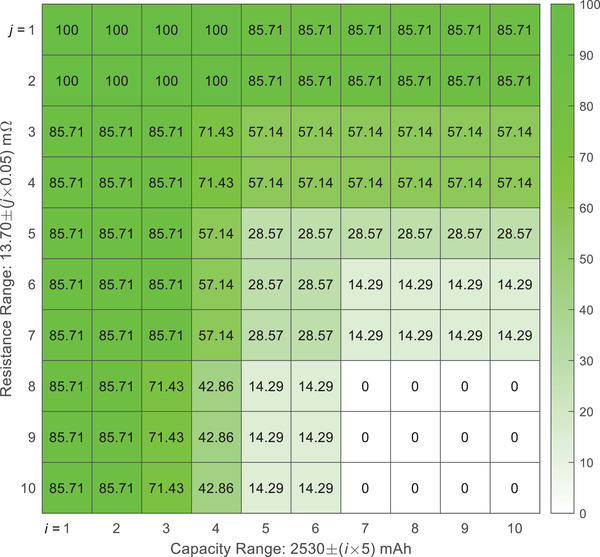
True positive rate (in %) of the predictions with different capacity and resistance ranges. For instance, the value in the 2^nd^ row 8^th^ column is 85.71, which means when *i* = 2 and *j* = 8, the true positive rate of the prediction is 85.71%.

**Figure 3:**
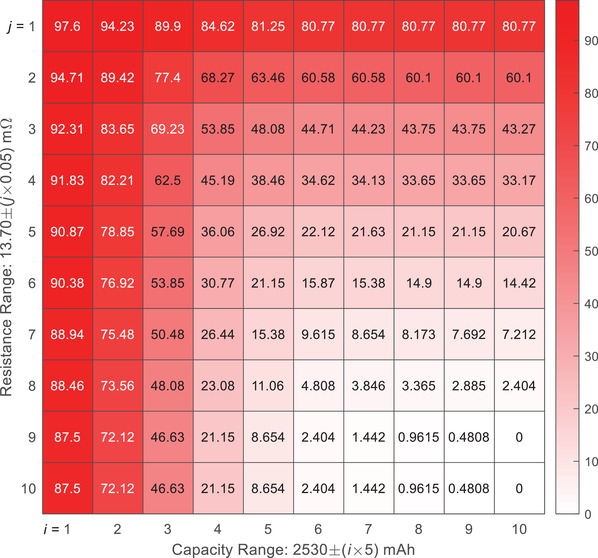
False negative rate (in %) of the predictions with different capacity and resistance ranges. For instance, the value in the 2^nd^ row 8^th^ column is 60.1, which means when *i* = 2 and *j* = 8, the false negative rate of the prediction is 60.1%.

In the CR method, the classification relies on two parameters ‐ capacity (*C*) and resistance (*R*). A battery is classified as normal if these two parameters fall into a certain tolerance range. For instance, when the allowable parameter ranges are selected as *C* ∈ [2.48, 2.58] Ah and *R* ∈ [13.2, 14.2] *m*Ω, the CR method will report all batteries as “normal”. In other words, all the abnormal individuals shown in Figure 1a cannot be identified, and both the true positive rate (TPR, rate of successfully identifying the abnormal batteries) and the false negative rate (FNR, rate of reporting normal batteries as abnormal) are 0%. By further narrowing down the parameter ranges, the TPR could improve at the cost of an increase in FNR. As shown in **Figures** **2** and  **3**, when the ranges are tuned so that the CR method can identify all abnormal batteries (TPR = 100%), the minimum FNR is 68.27%, implying that at least 68.27% of the normal batteries are predicted to be abnormal. On the other hand, when the FNR is controlled to be lower than 10%, the highest TPR is only 28.57%, implying that more than 70% of the abnormal batteries cannot be identified. From these results, even with the fine‐tuned parameters, the performance of the CR method may still not be satisfactory.

### 2.3 Identifying Lifetime Abnormality via Few‐Shot Learning Network

**Figure 4:**
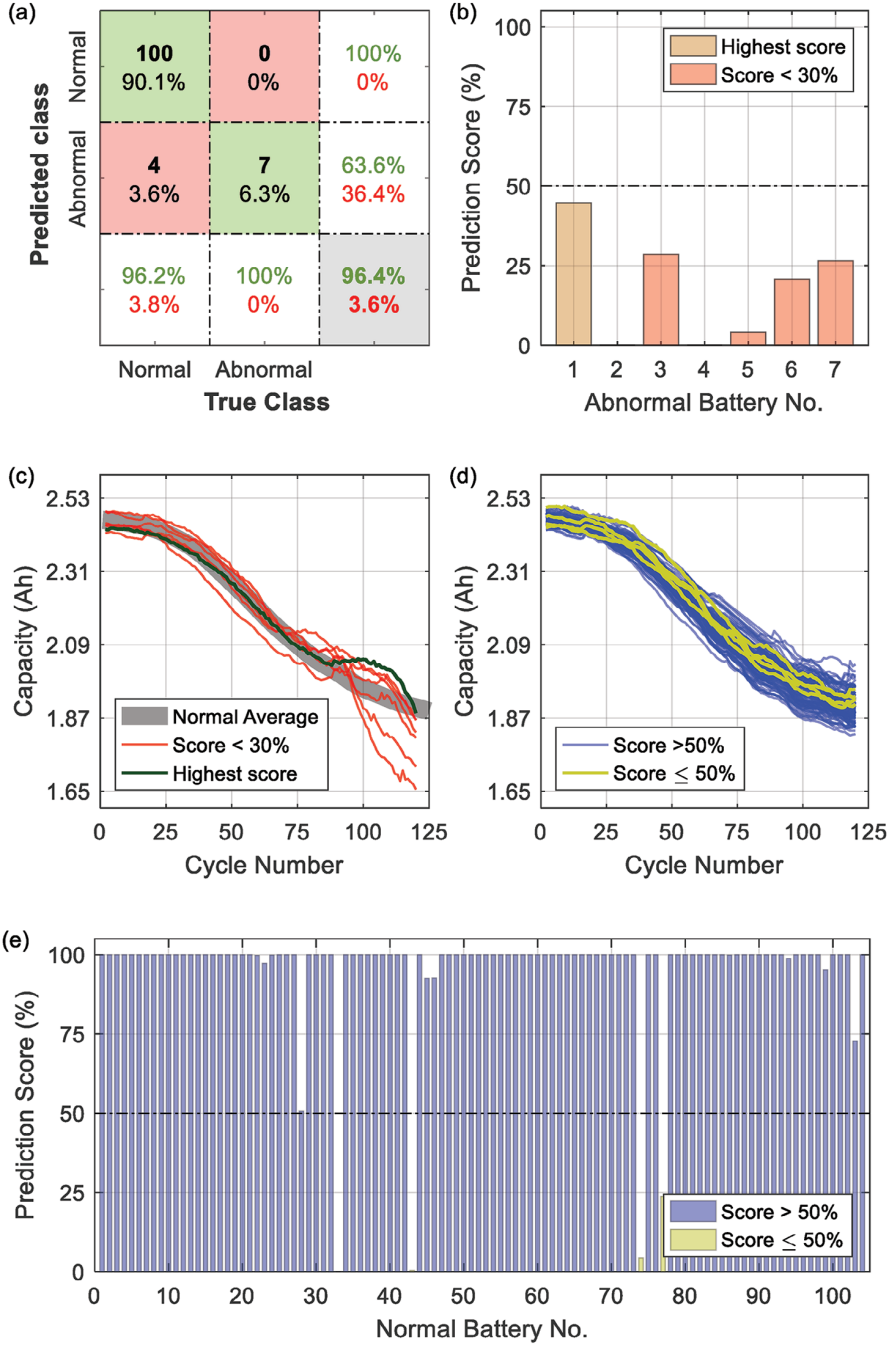
Classification results of the proposed method. a) Confusion matrix; b) Prediction scores of the abnormal batteries (the lower, the better); c) Aging trajectories of the abnormal batteries, where the one with the highest prediction score is highlighted with dark green; d) Aging trajectories of the normal batteries, in which the four batteries with the prediction score <50% are highlighted with yellow; e) Prediction scores of the normal batteries (the higher, the better).

The overall performance of our few‐shot learning network is summarized in **Figure** **4**a, in which 7 abnormal and 104 normal batteries are utilized for testing (See Section 4.1 for details). We can accurately screen out all seven abnormal batteries. In addition, 100 out of 104 normal batteries could be identified. The overall prediction accuracy achieves 96.4%, and the false alarm rate is only 3.8%. As an effective statistical index considering both the precision and recall of a model, the F_2_‐score (see Section 4.1 for details) of our prediction achieves 89.74%.

More specifically, the prediction scores (see Section 4 for details) of all abnormal batteries are given in Figure 4b. The scores of all batteries are lower than a predefined threshold, i.e., 50% in this work, implying that all abnormal batteries are accurately predicted to be “abnormal”. In our test, the first abnormal battery has the highest score (44.6%), and its aging trajectory is given in Figure 4c. Compared with other abnormal batteries, its average aging rate between the 90^th^ and 120^th^ cycle is indeed the lowest.

**Figure 5:**
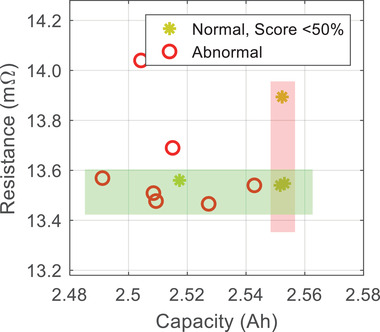
Comparison between the abnormal batteries and the normal batteries with prediction score <50%.

For the normal batteries, their prediction scores are given in Figure 4e. 100 out of 104 batteries receive a score >50%, implying that these batteries could be classified as “normal”. Further, the scores of 93 batteries are higher than 99%, indicating that our predictions are highly confident. For the false‐alarmed batteries with a score <50%, their aging trajectories are given in Figure 4d, and their resistance versus capacity plot is given in **Figure** **5**. The resistance of 5 out of 7 abnormal batteries lies between 13.45 and 13.57 mΩ, while 3 out of 4 false‐alarmed batteries also have this resistance range. In addition, the capacity of 3 out of 4 false‐alarmed batteries lies in the range of 2.551 to 2.553 Ah. In short, these false‐alarmed batteries are somehow similar to the abnormal ones, posing challenges to our few‐shot learning‐based classifier.

### 2.4 Discussion

#### 2.4.1 Results of Other Commonly Seen Algorithms

To further illustrate the superiority of the proposed method, we test the classification performance of six commonly used abnormal detection algorithms, including the one‐class supporting vector machine, auto‐encoder, density‐based spatial clustering of applications with noise (DBSCAN), isolation forest, K‐nearest neighbor, and local outlier factor model. The detailed results of these algorithms are given in Sections S.2.1–S.2.6, (Supporting Information). In our tests, the best conventional algorithm could only achieve an F_2_‐score of 37.8%, with an accuracy of 83.7% and a false alarm rate of 15.9%. Such a performance is significantly lower than the proposed method and cannot meet the general engineering requirements.

As detailed in Section 4, the proposed few‐shot learning is a supervised learning algorithm. The benefit of supervised learning is that it has a clear training target, resulting in more effective training processes. However, in an abnormality detection problem, we are unlikely to cover all possible abnormal cases in the training set, posing challenges to the generalization of our method. In our solution, we design networks that tell if any two batteries in the pool come from the same group, rather than directly telling if a battery is normal or not. In this way, we stand a chance to produce an effective classifier. From the posterior point of view, our method outperforms six main‐stream unsupervised abnormality detection algorithms. We use the minimum possible input requirement (the first cycle data only) and achieve the top‐class lifetime abnormal detection performance on the largest known dataset.

#### 2.4.2 Results of Different Cell Systems

**Figure 6:**
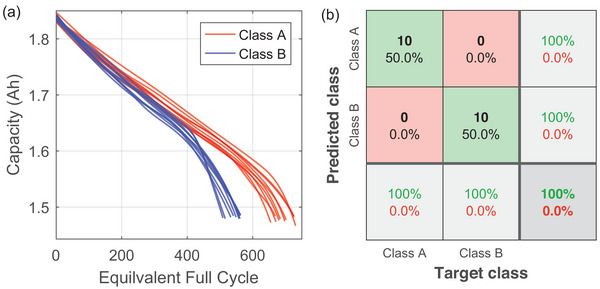
Additional results. a) Aging trajectories of different batteries; b) Confusion matrix of the prediction.

The battery's behavior changes significantly with chemistry and load profiles.^[^ ^25^ ^]^ To examine the effectiveness of the proposed framework for various cell systems with different testing procedures, an additional dataset from Ref. [26] was used. First, ten batteries with the highest life cycle and another ten with the lowest life cycle were first selected and then filled into two classes. The aging trajectories of these 20 batteries are shown in **Figure** **6**a. Then, our algorithm is carried out to implement the classification, with the results given in Figure 6b. In this dataset, the overall accuracy achieves 100%. Such a high accuracy indicates that the proposed framework can be easily used for different battery types and duty cycles. At the same time, conventional unsupervised methods listed in Section 2.4.1 can achieve only limited accuracy. The best result has an F_2_‐score of 86.5% and an accuracy of 71.4% only. The full details about the data and validation results can be found in the Section S.2.7 (Supporting Information).

It is worth noting that there is no well‐established theory that can accurately and physically explain how the battery's aging behavior over its lifetime is related to attributes measured at the beginning of life. However, the aging‐related attributes can be statistically calculated. According to Ref. [27], the top ten attributes are mostly associated with the battery's pulse resistance or electrochemical impedance spectroscopy (EIS) for batteries in Ref. [26]. As confirmed by Ref. [28] and our recent work,^[^ ^29^ ^]^ neural networks are capable of extracting EIS and general resistance information from the battery's operating data (e.g., constant‐current‐constant‐voltage charging data, see Section 4 for details). Therefore, it can be inferred that a powerful data‐driven machine can build a pathway from the first‐cycle testing data to the battery lifespan in‐explicitly.

## 3 Conclusion

Early‐stage lifetime abnormality prediction is critical to prolonging the service life of a battery pack, but technically challenging due to not only the limited information to be possibly extracted in the first few cycles but also the inherently low rate of battery abnormality. In this paper, we use the few‐shot learning method to predict the lifetime abnormality of the batteries with only first‐cycle aging data. Verified with the largest known dataset generated in this work, the proposed method successfully identifies all abnormal batteries, with a false alarm rate of only 3.8%. Our method cannot only be used on new batteries but also unlock exciting future research opportunities to assist in the screening of retired batteries to facilitate their second‐life usage, further extending the battery's service life. Given the limited experimental resources, the dataset used in this work was generated with accelerated aging tests for only one battery type (although it is the largest known dataset). When using the results in this work for highly diverse usage scenarios associated with different aging mechanisms or even different batteries, model migration or transfer learning techniques could be a solution to maintain high accuracy and robustness. The first‐cycle data used in this work will also be extended to the formation data so that the proposed method can be used by battery manufacturers without carrying out additional tests.

## 4 Experimental Section

### Overview

**Figure 7:**
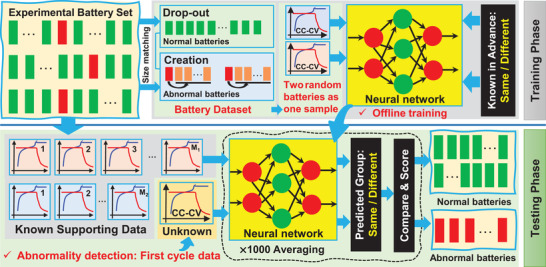
The framework of the proposed method.

The proposed battery lifetime abnormality detection method was a supervised data‐driven algorithm based on few‐shot learning, and it basically had two steps – training and testing. The overall scheme of the algorithm is given in **Figure** **7**. In the training phase, a group of 1000 neural networks was first trained with known data collected through the experiment. In the testing phase, these well‐trained networks could be utilized to predict whether the lifetime of an unknown battery was normal. Technical details are provided in the following subsections.

### Training Phase

In the training phase, it trained such networks that tell if any two randomly selected batteries belong to the same group. As described in **Table** **1**, when any battery *i* and battery *j* are both normal or abnormal, the network was expected to provide a “true” response. At the same time, if one battery was normal while the other was abnormal, the network was expected to provide a “false” response.

**Table 1:** Desired output of the few‐shot network.

	Battery *i*: normal	Battery *i*: abnormal
Battery *j*: normal	[1;0] (true)	[0;1] (false)
Battery *j*: abnormal	[0;1] (false)	[1;0] (true)

To implement the above‐mentioned functions, the input of the network should consist of the data collected from two batteries:

(1) $$IN={\left[\Gamma^{i},\Gamma^{j}\right]}^{T}$$

where Γ^*i*^ and Γ^*j*^ are column vectors containing the key features of the *i* ^th^ and *j* ^th^ battery, respectively.

To maximize the data mining capability of the neural network, the battery feature here was directly obtained from the raw data of the experiments. Specifically, the voltage data measured in the constant current charging phase and the current data measured in the constant voltage charging phase were combined as a column vector to serve as the input:

(2) $$\Gamma^{k}={\left[V_{1}^{k},\;V_{2}^{k}\;,\text{…},\;V_{L_{1}}^{k},I_{L_{1}+1}^{k},I_{L_{1}+2}^{k},\text{…},{I_{L}}_{2}^{k}\right]}^{T}$$

where *L*_1_ is the duration for the constant current phase, and *L*_2_ is the duration of the entire charging process. For the considered batteries and charging profile, we have *L*_1_ = 65 and *L*_2_ = 100.

Finally, a three‐layer neural network was employed to map the input to the output. The hidden layer of the network was activated by the *radbas*() function:

(3) $$radbas\left(x\right)=e^{-x^{2}}$$

and the output layer was activated by the *softmax*() function:

(4) $$y_{k}=\;softmax\left(z_{k}\right)=\frac{e^{z_{k}}}{\sum_{j=1}^{N}e^{z_{j}}}$$

where *Z* = [*z*_1_, *z*_2_, ⋅⋅⋅, *z*_*N*_]^*T*^ is the input of the softmax function, *N* is the number of output neurons, *z*_*j*_ ∈ *Z*, *z*_*k*_ ∈ *Z*, *j* ∈ [1, *N*], and *k* ∈ [1, *N*]. Following Table 1, *N* = 2 is selected in this work. The network's output is binarized with the following rule: we have [*y*] = 1 if 0.5 ⩽ *y* ⩽ 1, and [*y*] = 0 if 0 ⩽ *y* < 0.5.

Our network was trained by a conjugate gradient backpropagation algorithm with Fletcher‐Reeves updates,^[^ ^30^ ^]^ and full details are provided in the uploaded code. To minimize the influence of the random network initialization,^[^ ^31^ ^]^ we trained 1000 networks in the training phase and used their averaged output to compensate for the random errors in the testing phase (as detailed in the Section 4).

### Testing Phase

In the testing phase, we basically use the well‐trained networks to tell if an unknown battery from the testing dataset and a known battery (also named the “supporting battery” here) are in the same group. In other words, we test if the network's output corresponding to *IN* = [Γ_*u*_, Γ_*s*_]^*T*^ is “true”, where Γ_*u*_ is the feature vector extracted from the unknown battery, while Γ_*s*_ is the feature extracted from the supporting battery. As detailed in **Table** **2**, an unknown battery can be classified as “normal” if it is in the same class as a normal battery or in a different class with an abnormal battery.

**Table 2:** Prediction result for the unknown battery when the network's input is *IN* = [Γ_*u*_, Γ_*s*_]^*T*^.

	Network's output: [0;1] (false)	Network's output: [1;0] (true)
(Known) Supporting battery: abnormal	Normal	Abnormal
(Known) Supporting battery: normal	Abnormal	Normal

When we have a well‐trained network, we may be willing to use *M* supporting batteries (with *M*_1_ known normal samples and *M*_2_ known abnormal samples, *M* = *M*_1_ + *M*_2_, *M*_1_ > 0, *M*_2_ > 0, *M* ⩾ 2) to improve the reliability and robustness of our prediction. In this case, we can carry out *M* predictions with all the supporting batteries. For each time the unknown battery is predicted to be normal by a normal supporting battery, we add 1/*M*_1_*50% score to its rate; for each time the unknown battery is predicted to be normal by an abnormal supporting battery, we add 1/*M*_2_*50% score to it. After carrying out all predictions, the unknown battery is believed to be normal if its final score is greater than 50%.

We may also use the averaged prediction results of more networks to further minimize the random prediction error. When we have *H* (*H* ⩾ 1) well‐trained networks, the abnormality detection for an unknown battery can then be carried out for *H* times. For each time the unknown battery is predicted to be normal, we add 1/H*100% score to this battery's rate. Again, the unknown battery is predicted to be normal if the prediction score is greater than 50%. As pointed out in Section 4, we select *H* = 1000 in this work.

### 4.1 Data Preparation for Network Training

As a common issue for most abnormality detection problems, the percentage of abnormal samples was usually significantly lower than the normal ones. Therefore, a delicate data preparation method for network training was required. The data preparation method contains three parts, namely, data segmentation, normal data drop‐out, and abnormal data creation.

*Data‐Segmentation*

Given that it had only seven abnormal batteries in the dataset, the leave‐one‐out method^[^ ^32^ ^]^ was adopted for these batteries. Specifically, when training the network, it selected data from six abnormal batteries for training and leave only one for testing. This process was repeated seven times so that all abnormal batteries could be tested one time, and the seven sub‐tests were combined to provide the final results. For the 208 normal batteries, it simply used the first half for training and the remaining half for testing.

*Drop‐Out of Normal Samples*

With the above configurations, it had more than a hundred normal batteries but only six abnormal ones in the training set. Consequently, the training set was heavily unbalanced, posing challenges to accurate predictions.^[^ ^33^ ^]^ In this case, the drop‐out technique was employed to remove some normal batteries from the training set. To be specific, for each abnormal battery in the training set, we check its aging trajectory and find the N_1_ normal batteries whose aging trajectories were closest to it. Here the distance between two aging trajectories is defined by:

(5) $$D_{i,j}=\frac{1}{L}\sqrt{\sum_{k=1}^{L}{\left(C_{i,k}-C_{j,k}\right)}^{2}}\;$$

where *D*_*i*, *j*_ is the distance between the trajectories of the *i* ^th^ battery and the *j* ^th^ battery, *C*_*i*, *k*_ is the capacity of cell *i* measured at *k* ^th^ cycle, and *L* is the total number of the cycles evaluated. By selecting a suitable *N*_1_ (*N*_1_ = 3 is selected in this work), a large number of normal batteries could be removed from the training set. In addition, the remaining normal samples could be regarded as the “boundary” to classify the normal and abnormal batteries, which could improve the generalization of the network.^[^ ^34^ ^]^

*Creation of Abnormal Samples*

In addition to the unbalanced proportion, the low absolute number of abnormal batteries was also an important issue to handle. Here, we proposed to solve this issue by “creating” more abnormal data. The aim of this work was to use the data collected from the first cycle of the aging test to identify the lifetime abnormality. However, as shown in Figure 1 and many other battery aging datasets,^[^ ^22^, ^35^, ^36^ ^]^ the battery's behaviors in the first few cycles were highly similar. Therefore, we proposed to use the data collected in the first *N*_2_ cycles to enrich the training set. In other words, when we had actually collected one abnormal battery, we could pretend that we had collected *N*_2_ abnormal (fake) batteries. The “first cycle data” for these *N*_2_ fake batteries were obtained from the data of the abnormal battery collected from cycle 1 to cycle *N*_2_. In short, for each abnormal battery collected, it generated *N*_2_ feature vectors (Γ) in the training phase.

There were some issues worth pointing out. First, the strategy here was to “create” data from the experimental facts. It was different from the conventional resampling‐ or oversampling‐based “data‐creating” approaches, where rule‐based methods were basically used to draw random particles around the known positive samples.^[^ ^33^ ^]^ Second, the data‐creating method here was also applied to the selected normal batteries, to minimize the influence of slight battery aging during the period between cycle 1 and cycle *N*_2_. Third, the data creation was only applied in the training phase. In the testing phase, our method only compares the first cycle data of an unknown battery with the known data in the supporting set to implement the classification. Finally, for the specific dataset generated in this work, *N*_2_ = 4 is selected.

### Performance Indicators

Some terminologies and key performance indicators used in this work were introduced. First, the definitions of true positive, true negative, false positive, and false negative are given in **Table** **3**. Then, the accuracy of the prediction can be calculated by:

(6) $$\mathrm{Accuracy}=\frac{TP+TN}{TP+TN+FP+FN}\times 100\%$$

**Table 3:** Definition of the terminologies.

	Actual class: abnormal	Actual class: normal
Predicted class: abnormal	True Negative (TN)	False Negative (FN)
Predicted class: normal	False Positive (FP)	True Positive (TP)

The Precision and Recall can be defined respectively as:

(7) $$\mathrm{Precision}=\frac{TP}{TP+FP}\times 100\%$$

(8) $$\mathrm{Recall}=\frac{TP}{TP+FN}\times 100\%$$

The *F*_β_ score of the prediction is defined as:

(9) $$F_{\beta }=\left(1+\beta^{2}\right)\cdot \frac{\mathrm{Precision}\cdot \mathrm{Recall}}{\beta^{2}\cdot \mathrm{Precision}+\mathrm{Recall}}\times 100\%$$

When β = 1 is selected, Precision and Recall are given the same weight. When β < 1, Precision is more important. When β > 1, Recall becomes more important. β = 2 is utilized in this work since the consequences of missing alarms were more severe than that of the false alarm in battery lifetime abnormality detection.

## Conflict of Interest

The authors declare no conflict of interest.

## Supporting information

Supporting Information

## Data Availability

The code and data can be downloaded from: https://drive.google.com/drive/folders/1wMoXu‐G9zC6CPyo‐t742XNEkHYbPnWmb?usp=sharing
