# Supplementary material for: Detecting Abnormality of Battery Lifetime from First‐Cycle Data Using Few‐Shot Learning
Source: Adv Sci (Weinh). 2023 Dec 11;11(6):2305315. doi: 10.1002/advs.202305315 (PMC10853708; doi:10.1002/advs.202305315)
Supplement: Supplementary file 1 — Supporting Information [file ADVS-11-2305315-s001.pdf]

ADVANCED SCIENCE
Open Access

Supporting Information

for *Adv. Sci.*, DOI 10.1002/advs.202305315

Detecting Abnormality of Battery Lifetime from First-Cycle Data Using Few-Shot Learning

Xiaopeng Tang, Xin Lai, Changfu Zou, Yuanqiang Zhou, Jiajun Zhu, Yuejiu Zheng and Furong Gao**

S1. Data Acquisition

In total, 224 batteries of type FST-25M-18650 are tested to investigate the characteristics of their lifetime. These batteries are produced by Far East Smart Energy. Their nominal capacity is 2500 mAh, and the internal reference number (given by the manufacturer for the purpose of categorizing different battery types) is PS-RD-044. The photo of the batteries is shown in Figure S1.

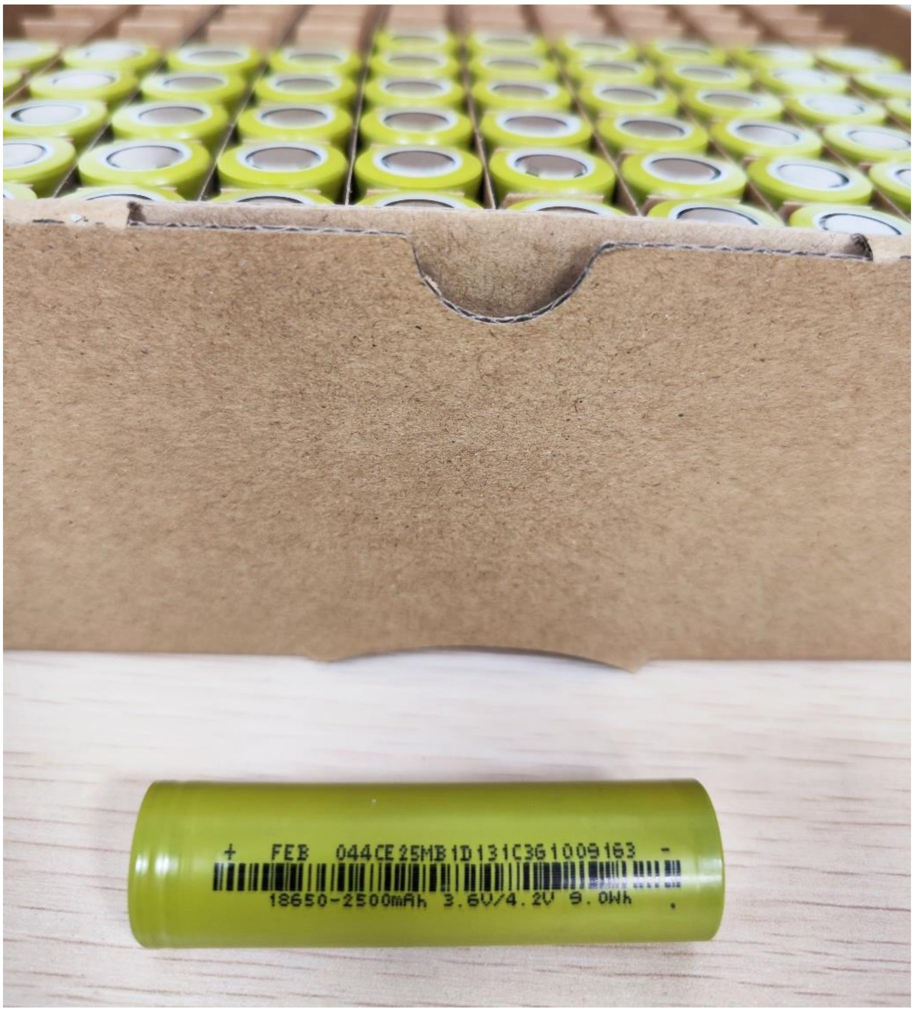

Figure S1. Photo of the selected battery.

For each battery, the resistance is first tested. Then, the batteries' initial capacity values are measured. Finally, an accelerated aging test is carried out for these batteries.

When testing the resistance, the batteries are first settled under a stable room temperature of 25±2℃ for at least 12 hours, and their open-circuit-voltage all lies within the range of 3621.5±2.5 mV, as illustrated in Figure S2(a). Then, the resistance is measured. For cells #01~#64 and cells #97~#224, the resistances are measured with HK3563D battery impedance tester, while for cells #65~#96, the resistances are

measured with CN0510-EVAL-AD5941BATZ impedance tester. The resistance values are all measured under 1kHz, and their distributions are shown in Figure S2-(b).

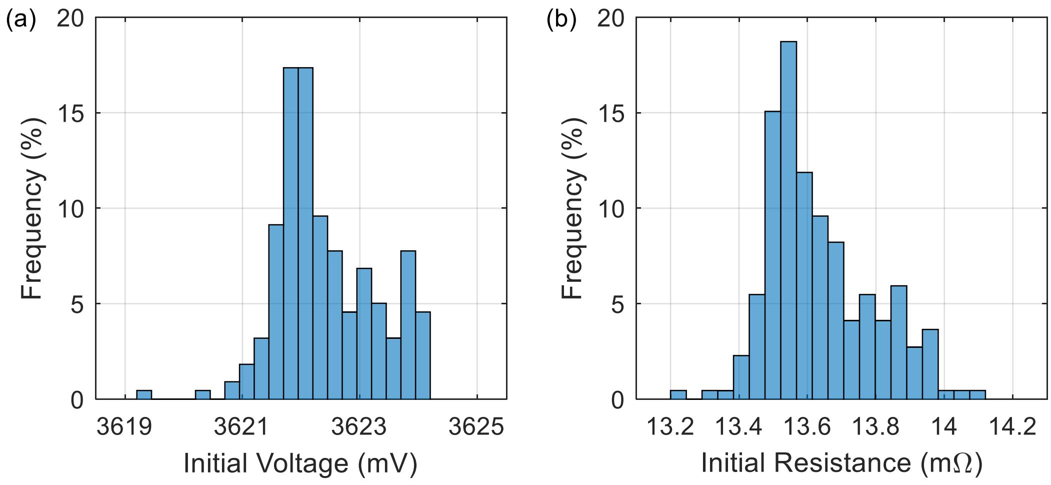

Figure S2. Distributions of the initial voltage and the initial resistance.

For batteries' initial capacities, their values are measured with the UPower battery testing system. The constant current-constant voltage (CCCV) profile is first used to fully charge the battery, followed by a rest period of 2 hours. Then, the constant current (CC) profile is adopted to fully discharge the batteries, followed by another 2-hour waiting profile. Finally, the batteries are fully charged again to support the follow-up tests. In the CC phase, the charging/discharging current is set to be 500 mA. The cut-off voltages are 4200 mV and 2750 mV for charging and discharging, respectively. The cut-off current is 250 mA in the CV phase. All tests are carried out under a stable room temperature of 25±2℃, and the data is recorded once per second. The capacity measured in the discharging phase is recorded as the battery's initial capacity, and the distribution is given in Figure S3 (a).

Regarding the accelerated aging experiments, they are implemented with, again, our UPower battery testing system. The CC profile is first applied to fully discharge the batteries, followed by a rest period of 10 minutes. The CCCV profile is then carried out to fully charge the batteries, and another 10-minute resting is then applied. Here, the current in the CC phase becomes 7500 mA while the cut-off conditions remain unchanged. All tests are carried out under a room temperature of 25±2℃, and the data is recorded once per 15 seconds to save storage. The capacity distribution of the first cycle is given in Figure S3 (b). The data measured in the first 120 cycles are utilized in this work, in which period the batteries' average capacity (measured with the accelerated aging test) drops from 2465 mAh to 1900 mAh, exhibiting a decrease of >20%.

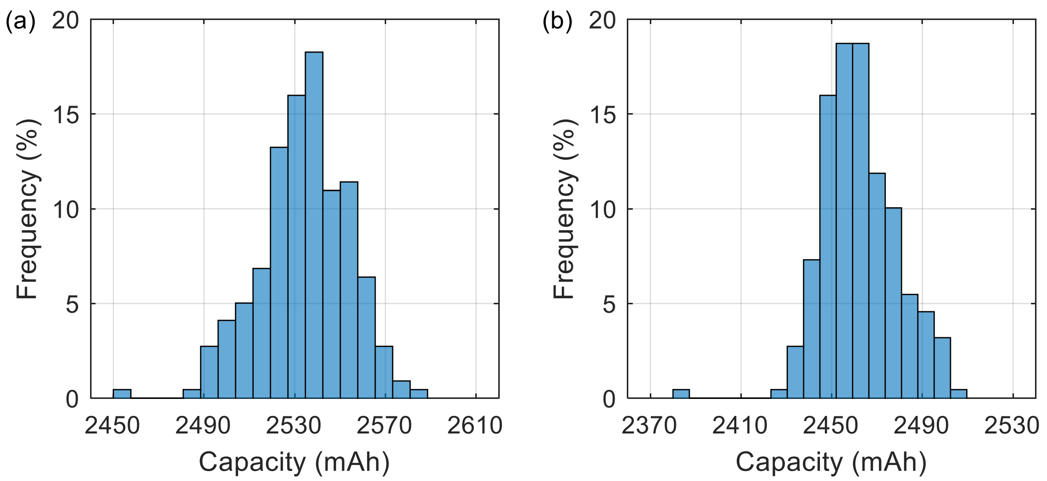

Figure S3. Distribution of the initial capacities. (a): Initial capacities tested with a constant current of 500mA; (b): First-cycle capacities of the accelerated aging test, whose current achieves 7500mA.

Given the limited testing capability of our lab, the aging tests are separately carried out in 7 groups, with 32 batteries for each group, as listed in Table S1. In the 5th group, there is an unexpected failure on channel #22, and the data is therefore abandoned. In the 7th group, an unexpected communication failure happens on channels #10~#13, and the data from these four channels is also abandoned. In addition, this communication failure influences the data recording of all remaining 28 batteries from the 70th cycle to the 86th cycle. The data of these 17 cycles is abandoned, while the remaining is kept.

In the remaining 219 out of 224 batteries, as shown in Figure S4, cell #175 and cell #219 have abnormal initial capacity values, while cell #51 and cell #83 have abnormal voltage trajectories in the first charging cycle of the accelerated aging test. These four batteries are also removed from our dataset because the abnormality that can be immediately observed is considered an easy problem in this work and not discussed. We focus on the lifetime abnormality, which can only be experimentally determined after a long time of battery usage. Finally, a dataset containing the lifetime behaviors of 215 batteries is generated. The cell number is also re-arranged from #1 to #215 in the dataset for clarity.

Table S1. Time arrangement for the tests.

Group No	Date
1	2022-05-07 – 2022-05-14
2	2022-05-18 – 2022-05-25
3	2022-05-26 – 2022-06-04
4	2022-06-09 – 2022-06-18
5	2022-06-22 – 2022-07-02
6	2022-07-04 – 2022-07-13
7	2022-07-15 – 2022-07-24

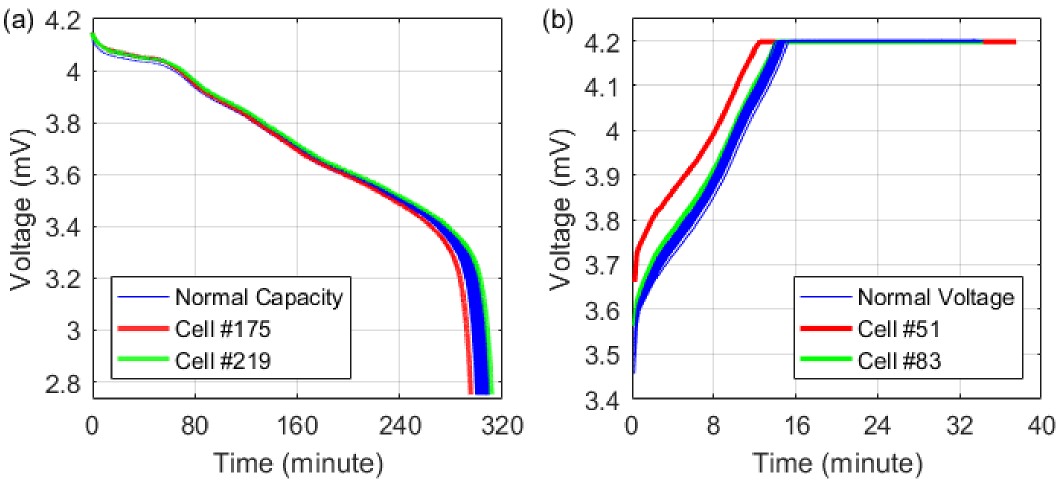

Figure S4. Initial battery screening results. (a): Abnormal initial capacities. (b): Abnormal initial voltage trajectories.

S2. Results of Other Commonly Seen Algorithms

In this section, six commonly used algorithms are utilized to detect abnormal batteries. The input features of these algorithms are exactly the same as the proposed methods, and their prediction results are given respectively in the following subsections. The purpose of this section is to carry out a comprehensive comparison among different methods, while the working principles of these widely used algorithms are omitted here for brevity. Full code for our implementations is provided according to the **Code and Data Availability Section**.

S2.1 One-class Supporting Vector Machine (OCSVM)

In this section, we use the first 50% of the normal batteries (104 groups of data) to train the one-class supporting vector machine (OCSVM). Then, its performance is tested against the remaining 104 normal and 7 abnormal batteries. The prediction results are shown in Figure S5. The overall prediction accuracy is 92.8%, however, the true positive rate is 0%. In other words, all abnormal batteries cannot be identified by the OCSVM algorithm.

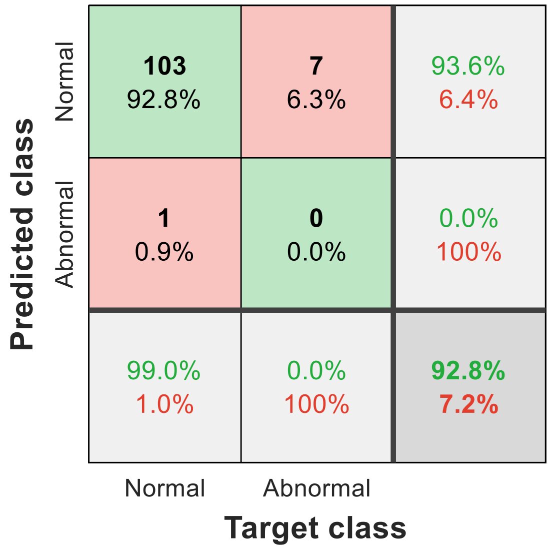

Figure S5. Confusion matrix of one-class supporting vector machine-based prediction.

S2.2 Auto-Encoder (AE)

In this section, we use the first 50% of the normal batteries (104 groups of data) to train the auto-encoder decoder model and test its performance on the other 104 normal and 7 abnormal batteries. The prediction results are shown in Figure S6. The overall prediction accuracy of the auto-encoder-based algorithm is 89.2%, and the F_2-score is 38.5%. More than 50% of the abnormal batteries cannot be identified.

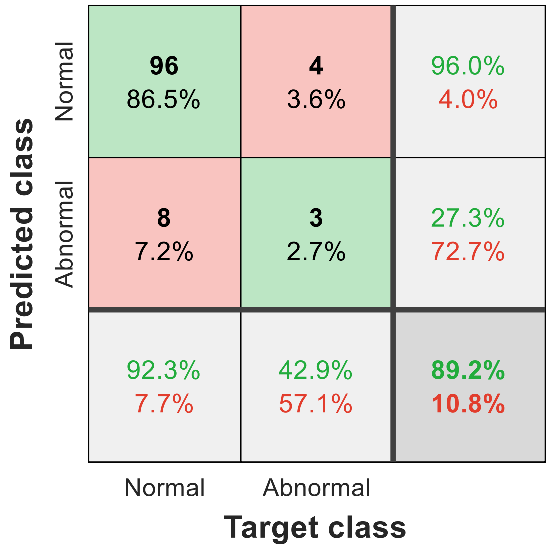

Figure S6. Confusion matrix of auto encoder-based prediction.

S2.3 DBSCAN

In this section, we use full data (208 normal and 7 abnormal batteries) to train the classifier and batteries that cannot be categorized into any clusters by the DBSCAN algorithm are reported as abnormal. The classification results are given in Figure S7. The overall prediction accuracy of the DBSCAN algorithm is 83,7%, and the F_2-score is 37.8%. More than 15% of the normal batteries are false alarmed.

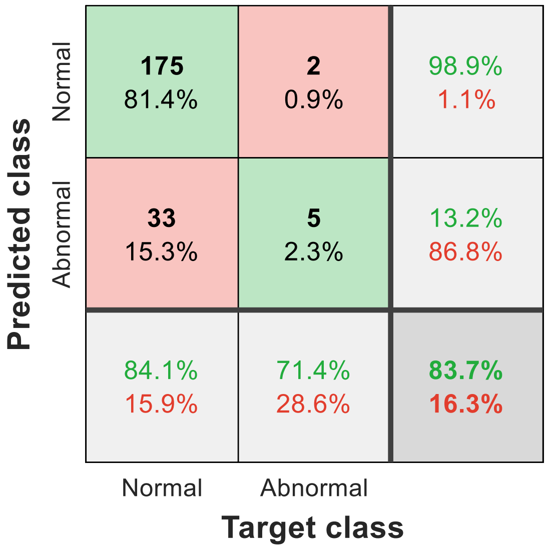

Figure S7. Confusion matrix of DBSCAN-based prediction.

S2.4 Isolation Forest (iso-Forest)

In this section, we use the first 50% of the normal batteries (104 groups of data) to train the isolation forest Then, its performance is tested with the remaining 104 normal and 7 abnormal batteries. The prediction results are shown in Figure S8. The overall prediction accuracy is 93.7%, however, the true positive rate is 0%. In other words, all abnormal batteries cannot be identified.

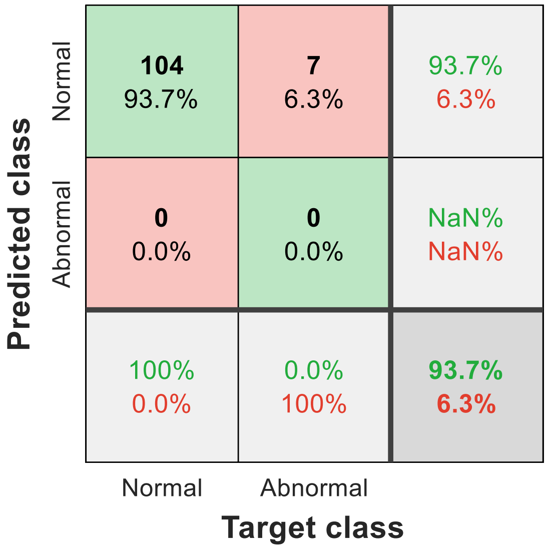

Figure S8. Confusion matrix of isolation forest-based prediction.

S2.5 K-nearest Neighbor (KNN)

In this section, we use full data (208 normal and 7 abnormal batteries) to train the classifier. For each group of data, the distances to its K-nearest neighbors are calculated. When the summation of these distances is higher than the threshold, the corresponding battery is reported as abnormal. The calculation results are given in Figure S9. The overall prediction accuracy achieves 92.6%, and the F_2-score is 34.9%. Here, more than 50% of the abnormal batteries cannot be identified.

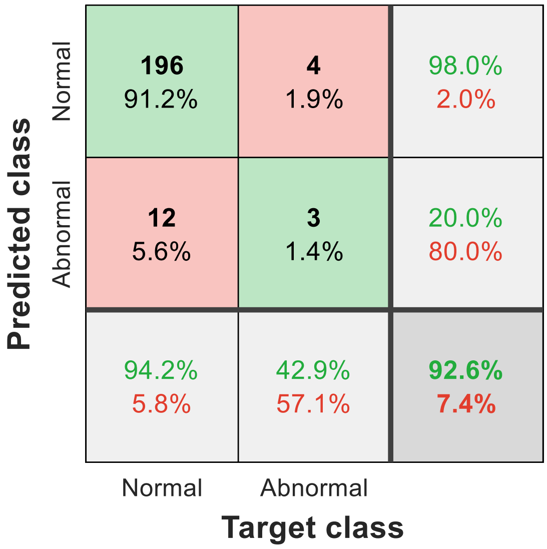

Figure S9. Confusion matrix of K-nearest neighbor-based prediction.

S2.6 Local Outlier Factor (LOF)

In this section, we use the first 50% of the normal batteries (104 groups of data) to train the local outlier factor model. Then, its performance is tested with the remaining 104 normal and 7 abnormal batteries. The prediction results are shown in Figure S10. The overall prediction accuracy is 93.7%, however, the true positive rate is 0%. In other words, all abnormal batteries cannot be identified.

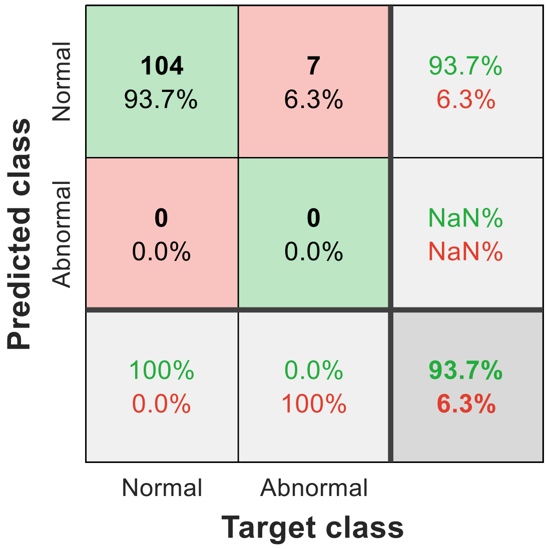

Figure S10. Confusion matrix of local outlier factor-based prediction.

S2.7 Results of other battery systems

In Section 2 of the main text, we used the dataset provided by RWTH Aachen University (Sanyo, 18650 type) to test if the proposed method can be used for different batteries. First, 10 batteries with the highest life cycle and another 10 with the lowest are selected and filled into two groups, respectively. Their battery numbers in the original dataset are [06, 07, 09, 12, 15, 16, 21, 30, 31, 41] for Class A (long life), and [18, 22, 24, 25, 26, 27, 28, 34, 35, 46] for Class B (short life), respectively. Before carrying out cyclic aging tests, initial constant-current-constant-voltage charging tests are conducted to determine the battery capacity. Following the technique of our paper, the voltage corresponds to the constant current phase and the current corresponds to the constant voltage phase are combined as an input vector for each cell. Here, the dimension of the feature is 267 for each cell, and they are plotted in Figure S11 (a-c).

The prediction scores are given in Figure S11 (d). In this dataset, our accuracy achieves 100%. At the same time, conventional unsupervised methods can achieve only limited accuracy, as listed in Table S2.

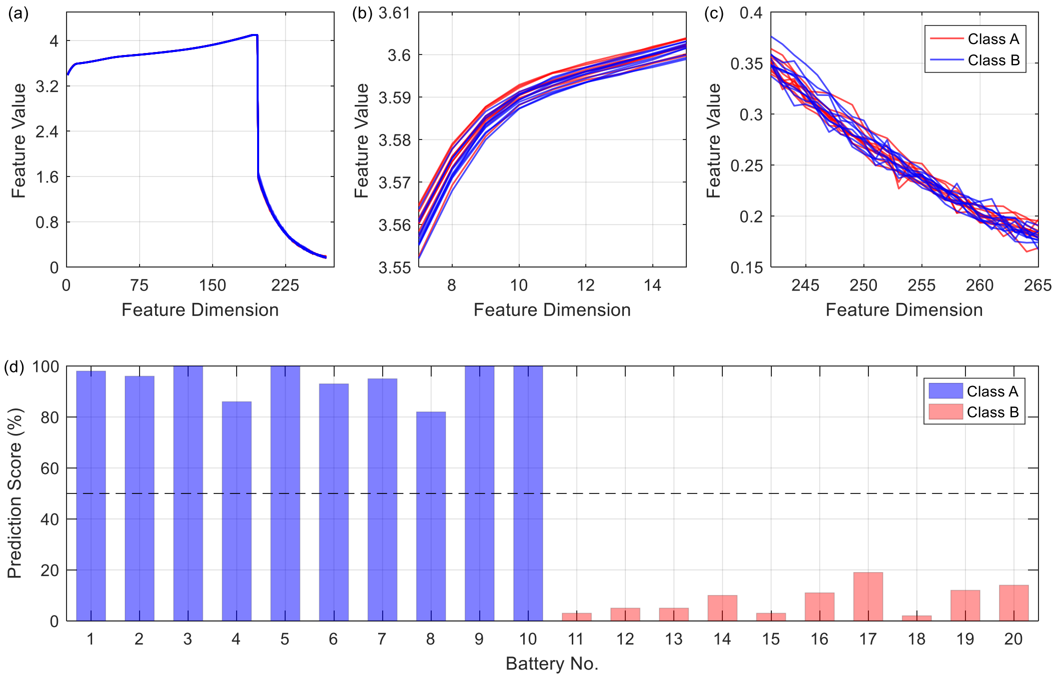

Figure S11. Additional results of the Aachen dataset. (a): Input features for Aachen dataset; (b-c): Zoom of (a); (d): Prediction scores for different classes, for Class A, the higher the better, while for Class B, the lower the better.

Table S2 Prediction accuracy and F2-score of different algorithms.

Method	OCSVM	LOF	KNN	iso-Forest	DBSCAN	AE
Accuracy	57.1%	28.6%	50%	28.6%	50%	71.4%
F2-score	45.5%	NA*	83.3%	NA*	22.7%	86.5%

*: No batteries in Class B can be accurately classified.
